# Low Levels of Antibody-Dependent Enhancement in Vitro Using Viruses and Plasma from Dengue Patients

**DOI:** 10.1371/journal.pone.0092173

**Published:** 2014-03-18

**Authors:** Panjaporn Chaichana, Tamaki Okabayashi, Orapim Puiprom, Mikiko Sasayama, Tadahiro Sasaki, Akifumi Yamashita, Pongrama Ramasoota, Takeshi Kurosu, Kazuyoshi Ikuta

**Affiliations:** 1 Mahidol-Osaka Center for Infectious Diseases (MOCID), Faculty of Tropical Medicine, Mahidol University, Bangkok, Thailand; 2 Center of Excellence for Antibody Research (CEAR), Faculty of Tropical Medicine, Mahidol University, Bangkok, Thailand; 3 Department of Virology, Research Institute for Microbial Diseases, Osaka University, Osaka, Japan; 4 JST/JICA, Science and Technology Research Partnership for Sustainable Development (SATREPS), Tokyo, Japan; University of Missouri, United States of America

## Abstract

**Background:**

The majority of dengue patients infected with any serotype of dengue virus (DENV) are asymptomatic, but the remainder may develop a wide spectrum of clinical symptoms, ranging from mild dengue fever (DF) to severe dengue hemorrhagic fever (DHF). Severe cases occur more often in patients who experience a secondary infection with a different virus serotype. A phenomenon called antibody-dependent enhancement (ADE) has been proposed to explain the onset of these severe cases, but the exact mechanism of ADE remains unclear.

**Methodology/Principal Finding:**

Virus neutralization and ADE assays were performed using ultracentrifugation supernatants of acute-phase sera from patients with secondary infections or human monoclonal antibodies (HuMAbs) as anti-DENV antibodies. Virus sources included infectious serum-derived viruses from the ultracentrifugation precipitates, laboratory-culture adapted DENV, or recombinant DENVs derived from patient sera. In contrast to the high levels of ADE observed with laboratory virus strains, low ADE was observed with autologous patient-derived viruses, when patient sera were used to provide the antibody component in the ADE assays. Similar results were obtained using samples from DF and DHF patients. Recombinant-viruses derived from DHF patients showed only minor differences in neutralization and ADE activity in the presence of HuMAbs or plasma derived from the same DHF patient.

**Conclusion/Significance:**

Serum or plasma taken from patients during the acute phase of a secondary infection showed high levels of ADE, but no neutralization activity, when assayed in the presence of laboratory-adapted virus strains. By contrast, serum or plasma from the same patient showed high levels of neutralization activity but failed to induce significant ADE when the assays were performed with autologous virus. These results demonstrate the significance of the virus source when measuring ADE. They also suggest that repeated passage of DENV in cell culture has endowed it with the capacity to induce high levels of ADE.

## Introduction

Dengue, a mosquito-borne infectious disease caused by four serotypes of dengue virus (DENV-1 to -4), is becoming more widespread in tropical and subtropical regions, posing an increasing global public health concern. DENV has a positive-sense, single-stranded RNA genome of approximately 11 kb that encodes a capsid protein (C), a pre-membrane protein (prM), and an envelope glycoprotein (E), in addition to seven nonstructural proteins, NS1, NS2A, NS2B, NS3, NS4A, NS4B, and NS5 [1].

Primary infection with any serotype of DENV establishes life-long immunity and protection against infection with the same serotype. However, the immune responses induced against one serotype of DENV do not protect against infection by other serotypes, allowing secondary infection with heterotypic DENV [2], [3]. Epidemiologic studies suggest that acute cases of dengue illness, including dengue hemorrhagic fever (DHF) and dengue shock syndrome (DSS), occur mostly among secondarily infected patients and cause severe symptoms [2], [4]-[6]. A hypothetical mechanism for this phenomenon has been proposed, called antibody-dependent enhancement (ADE), in which one viral serotype can use pre-existing non-neutralizing anti-DENV antibodies induced by previous infection with a different serotype to gain entry to Fc receptor-positive macrophages [7]. This immune enhancement would be a major impediment to the development of dengue vaccines. Epidemiological studies show that most DENV infections are either asymptomatic or lead to uncomplicated dengue fever (DF) [8], even among patients secondarily infected with a heterotypic DENV serotype [9]. Symptomatic cases appear in only 1% to 5% of infections; however, such cases often result in DHF [3]. Thus, ADE might not always be responsible for severe symptoms in dengue patients. An epidemiological study of a large number of infants born to DENV-seropositive mothers showed that, although ADE could be detected, it did not correlate with the incidence of DF and DHF [10].

Immunohistochemical studies of DENV-infected human tissues identified macrophages in lung, spleen and lymph nodes as major targets of DENV infection [11], [12]. Recent studies using quantitative RT-PCR and flow cytometry revealed the presence of positive strand DENV RNA and/or DENV antigens in a major cellular component of the peripheral blood mononuclear cell (PBMC) population, including monocytes, T/NK, B-cells and dendritic cells, with the highest amount found in B-cells [13]–[16]. Nonetheless viral RNA was equally distributed among different cell types in both DF and DHF patients, indicating that there was no preferential expansion of DENV in any particular cell type and, therefore, no role for anti-DENV antibodies in the expansion of discrete cell subsets.

Generally, *in vitro* ADE-related studies have used laboratory-adapted virus strains and murine and/or human monoclonal antibodies (MAbs) specific to viral surface proteins such as prM and E [17]–[23]. Recent clinical trials using a cocktail of yellow fever vaccine strains expressing both prM and E (prM-E) proteins derived from individual DENV serotypes (CYD-TDV; Sanofi Pasteur), demonstrated that no (or only minimal) ADE activity was induced *in vitro* when sera from vaccinated or control subjects were used at low dilutions [24], [25]. This suggests that the mechanism of ADE in experimental *in vitro* systems is different from that during infections in patients.

Here, we examined virus neutralization and ADE *in vitro* using viruses and antibodies obtained directly from the sera of patients suffering from acute secondary DENV infection without any passage in cells. We found that virus neutralization and ADE activities differed greatly depending on whether the viruses were derived from patients or were laboratory-adapted strains.

## Materials and Methods

### Cells and viruses

Vero cells were cultured at 37°C in an atmosphere containing 5% CO_2_ in minimum essential medium (MEM) (Sigma) supplemented with 10% fetal bovine serum (FBS; GIBCO BRL). C6/36 cells were cultured at 28°C (5% CO_2_) in Leibovitz's L-15 medium (GIBCO BRL) supplemented with 10% FBS and 10% tryptose phosphate broth (Sigma). Both cell lines were kindly provided by Dr. Prida Malasit (Faculty of Medicine Siriraj Hospital, Mahidol University). Erythroleukemia-derived K562 cells (semi-adherence-type [a courtesy of Dr. Eiji Konishi, BIKEN Endowed Department of Dengue Vaccine Development, Mahidol University] and suspension-type [Research Institute for Microbial Diseases, Osaka University]), and THP-1 cells were maintained in RPMI-1640 medium (Hyclone) supplemented with 10% FBS at 37°C (5% CO_2_). Suspension-type K562 cells were generally used in the ADE assays [10], [25], whereas semi-adherent cells were used for virus titration assays (since their properties are more suited to the assay systems) [26].

All cell lines used in this study were originally purchased from American Type Culture Collection (ATCC), except semi-adherent K562 [26] and suspension THP-1 cells [23], where selective passages were established to obtain the desired sub-populations.

Laboratory strains of DENV-1 16007, DENV-2 16681, and DENV-3 16562 were propagated in Vero cells. These Southeast Asian genotype viruses were originally isolated from DHF patients in Thailand (DENV-1 and -2) and the Philippines (DENV-3) [27]. Recombinant DENVs and DENV-2 strain 16681 (control) were propagated in C6/36 cells.

### Anti-DENV MAbs

Two hybridoma cell lines secreting anti-DENV human MAbs (HuMAbs), D30-3A1E2 and D30-1E7B8, were prepared by fusion of peripheral blood lymphocytes from a Thai DHF patient (D30) with a fusion partner cell, SPYMEG [28]. Hybridomas were cultured to confluency in DMEM supplemented with 10% FBS. The culture medium was then replaced with Hybridoma-SFM (serum-free medium, Life Technologies). HuMAb IgG_1_ was purified from the serum-free culture fluid of hybridomas by using HiTrap Protein G HP Columns (GE Healthcare, UK) as previously described [23]. The IgG concentration was measured using the Pierce BCA Protein Assay kit (Thermo Scientific). Murine MAb against DENV E, named 4G2 [29], was used to detect foci in focus-forming immunoassays.

### Serum samples from dengue patients

Blood samples were collected (without anticoagulant) from ten Thai dengue patients at the Hospital for Tropical Diseases (HTD), Faculty of Tropical Medicine, Mahidol University, Thailand. Samples were taken during the acute phase of infection (around 1 week after the onset of fever). In addition to clinical diagnosis, serum samples obtained from these patients were tested to confirm DENV infection using the *in vitro* immuno-chromatographic one step dengue NS1 antigen and IgM/IgG assay (SD BIOLINE Dengue Duo kit, SD, Kyonggi-do, Korea) according to the company’s protocol.

DENV virions in patient sera were collected by ultracentrifugation at 77,000× g for 30 minutes at 4°C in a Beckman Coulter Optima TLX-Ultracentrifuge equipped with a TLA 100.3 rotor. Pellets containing DENV virions were suspended in MEM and kept at −80°C until use. The supernatant containing anti-DENV antibodies were stored at −80°C until further use.

### Virus titration

Infectious titers of virus stocks were examined in focus-forming immunoassays in Vero cells [30] and K562 cells [26] and the results were expressed as focus-forming units (FFU). Briefly, monolayers of Vero cells or semi-adherent K562 cells grown in poly-L-lysine 96-well assay plate (BD Biosciences) were infected with DENV (serial 10-fold dilutions) and then incubated at 37°C (5% CO_2_). After 24 hours (for K562 cells infected with DENV-2), 48 hours (for K562 cells infected with DENV-1 and DENV-3), or 72 hours (for Vero cells), infected cells were fixed and immuno-stained with murine DENV-specific MAb 4G2 followed by anti-mouse secondary antibody. Dark brown spots of infected cells were developed by adding substrates. Infected cells were then visualized under microscope. The number of foci per well was counted to calculate virus titers.

### Reverse transcriptase-PCR (RT-PCR) for DENV serotyping

DENV serotypes in acute phase sera from dengue patients were determined by RT-PCR using primers specific for individual DENV serotypes, as previously described [31]. Briefly, viral RNA was extracted from serum samples using QIAmp viral RNA mini kit (QIAGEN) according to the manufacturer’s instructions. RNA was used as a template for cDNA synthesis using the Superscript III RT kit (Invitrogen, Carlsbad, CA). The first PCR reaction was performed with Taq DNA polymerase (Takara, Kyoto, Japan) using primers DEUL and DEUR (Table 1). The resulting amplicon was then subjected to nested PCR using primer sets D1L/D1R, D2L/D2R, D3L/D3R, and D4L/D4R to detect different DENV serotypes (Table 1).

**Table 1:** List of primers used in the study.

Primer name	Primer sequence	Length (bp)	Reference
DEUL	5′-TGGCTGGTGCACAGACAATGGTT-3′	23	31
DEUR	5′-GCTGTGTCACCCAGAATGGCCAT-3	23	31
D1L	5′-GGGGCTTCAACATCCCAAGAG-3	21	31
D1R	5′-GCTTAGTTTCAAAGCTTTTTCAC-3′	23	31
D2L	5′-GCTTAGTTTCAAAGCTTTTTCAC-3	23	31
D2R	5′-CCGGCTCTACTCCTATGATG-3	20	31
D3L	5′-CAATGTGCTTGAATACCTTTGT-3	22	31
D3R	5′-GGACAGGCTCCTCCTTCTTG-3′	20	31
D4L	5′-GGACAACAGTGGTGAAAGTCA-3′	21	31
D4R	5′-GGTTACACTGTTGGTATTCTCA-3	22	31
prMFw	5'-AACATCTTGAAYAGGAGACGCAG-3'	23	This study
NS1Rv	5'-CCAGCTCACRACGCAACCRCTATC-3'	24	This study
prM-EFw	5′-ATCATTATGCTGATTCCAACAGTGATGG-3′	28	This study
prM-ERv	5′-CTATCGGCCTGCACCATGACTCCCAAATAC-3′	30	This study
pmwR05Fw	5′-GTGAGCTGGAAAAACAAAGAACTG-3′	24	This study
pmwR05Rv	5′-TTCAAGATGTTCAGCATCCTTCC-3′	23	This study

### Preparation of anti-DENV antibodies from serum

Serum samples from dengue patients (HTDs) were ultracentrifuged at 77,000× g for 30 minutes at 4°C, and the supernatants containing anti-DENV antibodies were stored at –80°C until use [32]. IgM and IgG levels in the ultracentrifugation supernatants were determined using Dengue Virus IgM Capture DxSelect (OUS) and Dengue Virus IgG DxSelect (OUS), respectively, according to the manufacturer’s instructions (Focus Diagnostics, Cypress, CA).

### Generation of recombinant DENV cDNA clones

**Figure 1:**
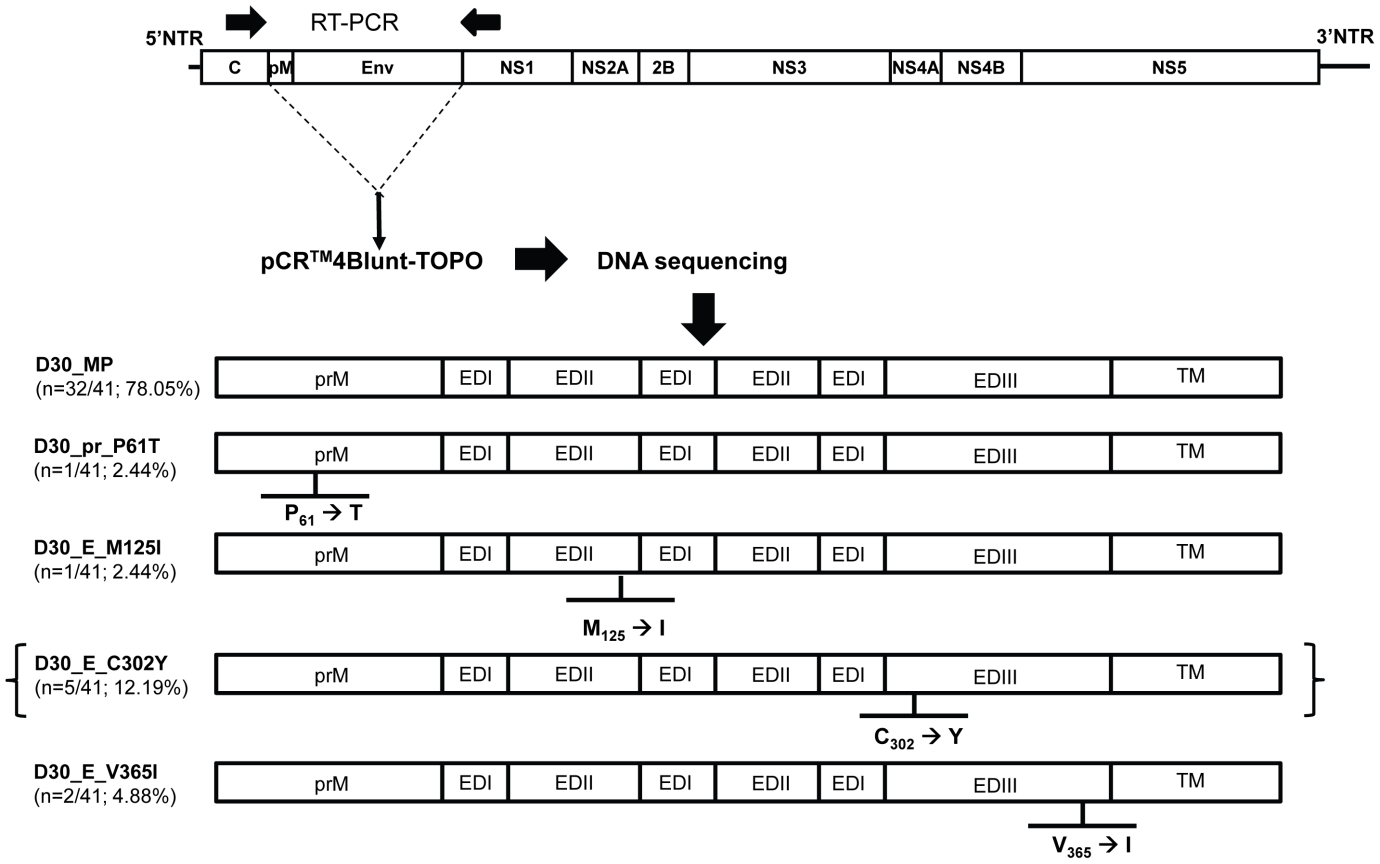
Construction of recombinant DENVs based on virus samples from patient plasma. RNA was extracted from the plasma of patient D30 and the prM-E encoding region of DENV was amplified by RT-PCR and cloned into the pCR4Blunt-TOPO vector for DNA sequence analysis. Representative prM-E region variants were selected and used to construct full-length DENV cDNA clones based on plasmid pmMW/R05-624. The resultant full-length cDNAs containing D30-derived variants were used as templates for RNA synthesis, and *in vitro*-transcribed viral RNAs were transfected into C6/36 cells. Supernatants from transfected cells were passaged once in C6/36 to obtain adequate quantities of viruses. EDI, envelope domain I. EDII, envelope domain II. EDIII, envelope domain III. TM, transmembrane. The recombinant DENV clone in brackets was excluded from further study due to an inadequate titer.

Viral RNA was extracted from the ultracentrifugation pellet of DHF patient (D30) acute-phase plasma using the QIAmp Viral RNA mini kit, as previously described [23]. To amplify the prM-E region of DENV, the viral RNA was subjected to RT-PCR using Primestar GXL DNA polymerase (Takara) and primers prMFw and NS1Rv (Table 1). The amplified product was cloned into the pCR4Blunt-TOPO vector (Invitrogen) and then transformed into competent *E. coli*. A total of 41 positive clones were selected for DNA sequencing at Macrogen Inc., Seoul, Korea. The nucleotide and amino acid sequences of the prM-E region were verified and aligned using BioEdit software. Subsequently, the prM-E encoding cDNA fragment of full-length infectious DENV-2 cDNA clone pmMW/R05-624 [30] was replaced by the corresponding cDNA fragments obtained from the individual clones (Figure 1). In brief, the prM-E region of the individual clones was amplified using primers prM-EFw and prM-ERv (Table 1). The pmMW/R05-624 plasmid was PCR amplified in parallel using the primers pmwR05Fw and pmwR05Rv (Table 1) to generate a linearized plasmid backbone lacking prM-E gene. Subsequently, the amplified products were subjected to In-Fusion reaction and then cloned into Stellar competent cells (Clontech). Resulting full-length cDNA clones were verified by sequencing. Plasmids with correct sequences were linearized and used as a template for RNA synthesis using the mMESSAGE mMACHINE kit (Ambion Inc, Austin, Tx). *In vitro*-transcribed viral RNA was transfected into C6/36 cells using Lipofectamine 2000 (Invitrogen). Supernatants from the transfected cells were collected and passaged once in C6/36 cells to produce recombinant virus stocks. Virus titers were determined in focus-forming immunoassays as described above and nucleotide sequences of the cloned prM-E regions were confirmed before further use.

### Virus replication kinetics

Overnight cultures of C6/36 and Vero cells at 80% confluence were infected with the recombinant DENV or the DENV-2 16681 control virus at a multiplicity of infection (MOI) of 0.001. After incubating for 2 hours, the cells were washed and incubated for 5 days in medium supplemented with 2% FBS. The culture fluids were harvested daily for virus titration in focus-forming immunoassays in Vero cells, as described above.

For infection of suspension-type K562 cells, 50 μl of virus suspension were mixed with K562 cell pellets at MOI of 0.1. After incubating at 37°C for 2 hours, the virus was removed by centrifugation and infected cells were washed twice with RPMI-1640 medium. The cells were then incubated for a further 4 days in RPMI-1640 medium supplemented with 2% FBS. Supernatants were harvested daily for virus titration in Vero cells.

### Virus neutralization and ADE assay

Heat-inactivated serum samples, D30-plasma, or purified HuMAbs were serially diluted 10-fold in RPMI-1640 medium and then mixed with virus solution at an MOI of 0.1 or 0.02. After incubating at 37°C (5% CO_2_) for 30 minutes, virus-antibody mixtures were added to K562 cells and incubated under the same conditions for another 2 hours before transfer into 48-well microplates. Following cultivation of the infected K562 cells for 3 days in maintenance medium supplemented with 2% FBS, both the fluid and the floating cell fractions were harvested. The fluid fractions from low-speed centrifugation (200× g for 5 minutes) were used for virus titration in focus-forming immunoassays in Vero cells.

This assay was also performed using THP-1 cells, as described previously [23]. The 10-fold serial dilutions of plasma were incubated with virus solution at 37°C for 30 minutes. THP-1 cells in serum-free medium were inoculated with these plasma-virus mixtures and incubated at 37°C for 2 hours. After adding RPMI-1640 medium supplemented with 2% FBS, the cells were incubated for a further 3 days at 37°C. Finally, the fluid fractions obtained after low-speed centrifugation were subjected to virus titration in focus-forming immunoassays using Vero cells, while total RNA was extracted from the cellular fractions using TRIzol reagent (Life technologies). The RNA was subjected to one-step quantitative (real-time) RT-PCR using the QuantiTect SYBR green RT-PCR kit (QIAGEN, Germany), according to the manufacturer’s instructions. The data derived from real-time RT-PCR were analyzed using the ΔΔCt analysis method [33]. GAPDH was used as an internal control. The assays were performed in triplicate and the results were expressed as the mean ± standard deviations (SD).

### Accession number

The prM-E sequences obtained from patient D30 were deposited in Genbank (http://www.ncbi.nlm.nih.gov/GenBank/) under accession numbers KF729018 - KF729022.

### Ethics statement

All human specimens were collected using research protocols approved by the Ethics Committee of the Faculty of Tropical Medicine, Mahidol University, and written informed consent was obtained from all participants.

## Results

### Patient serum samples showed low ADE and high levels of virus neutralization

**Figure 2:**
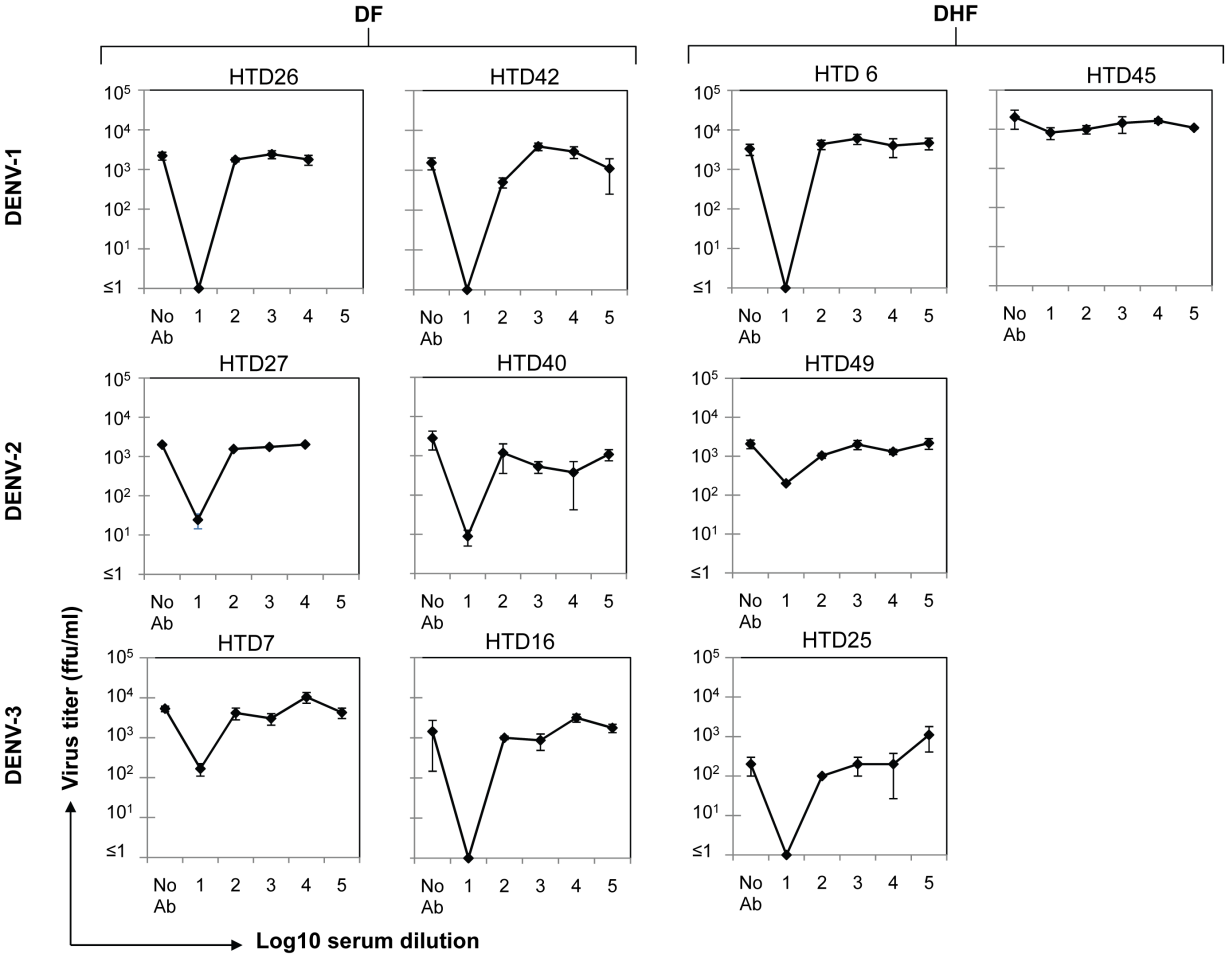
Low ADE when using serum and viruses from DENV-patients. Serum samples (DENV-1, DENV-2, and DENV-3) from HTD dengue patients with DF and DHF symptoms were ultracentrifuged to precipitate DENV virions which were used in assays without any subsequent passage in cells. The supernatant fractions were heat-inactivated at 56°C for 30 minutes and then serially diluted 10-fold. The dilutions were mixed for 30 minutes at 37°C with the precipitated virions from autologous plasma at an MOI of 0.02. The virus-antibody complexes were added to K562 cells and incubated for 2 hours at 37°C before the addition of maintenance medium supplemented with 2% FBS. The cells were then incubated for a further 3 days. Supernatants were harvested for virus titration by focus-forming immunoassay in Vero cells, and the results are expressed as FFU/ml. The mean ± SD of triplicate experiment is shown. ‘No Ab’ means virus infection in the absence of plasma. The ‘No Ab’ value was used as a baseline for calculating virus infection enhancement.

Initially, we examined virus neutralization and ADE induction using K562 as a host cell line. Anti-DENV antibodies and infectious DENV virions derived from the acute-phase serum of ten HTD dengue patients (six DF and four DHF patients) secondarily infected with DENV-1, -2, or -3 were used in the assays (Table 2). As shown in Figure 2, the titers of the different viruses from infected K562 cells in the absence of anti-DENV antibodies varied greatly, from around 10^4^ in sample HTD45 to around 10^2^ in sample HTD25, even though the K562 cells were infected at the same MOI. These results suggest that the susceptibility of K562 cells to DENV infection varied depending on the patient the virus sample was isolated from. High levels of virus neutralization (> x100–1000) were observed using samples from DF patients HTD26, HTD42, HTD27, HTD40, and HTD16, as well as samples from DHF patients HTD6 and HTD25. For the remaining samples, zero (HTD45, a DHF patient) or low levels (> ×100; HTD7, a DF patient; and HTD49, a DHF patient) of virus neutralization were observed. There was little or no evidence of ADE under these assay conditions when antibody and virus samples from any of the ten patients were used, regardless of virus serotype or disease severity.

**Table 2:** DENV serotypes, viremia titer, and anti-DENV antibody isotypes in DENV-infected patients.

			Viremia titers^d^	IgM^e^		IgG^f^		Virus infection^g^	
ID^a^	Symptom^b^	Serotype^c^	(ffu/mL sera)	Index	Interpretation	Index	Interpretation	IgM/IgG	Interpretation
HTD6	DHF	DV1	(7.72±1.84) ×10^5^	0.78	Neg	1.14	Pos	0.68	Secondary
HTD7	DF	DV3	(4.80±1.05)×10^5^	0.84	Neg	4.86	Pos	0.17	Secondary
HTD16	DF	DV3	(3.88±0.78)×10^4^	0.85	Neg	3.96	Pos	0.21	Secondary
HTD25	DHF	DV3	(1.08±0.12) ×10^5^	2.27	Pos	4.17	Pos	0.54	Secondary
HTD26	DF	DV1	(4.49±0.75) ×10^5^	0.59	Neg	2.97	Pos	0.20	Secondary
HTD27	DF	DV2	(4.52±1.07) ×10^5^	0.50	Neg	1.67	Pos	0.30	Secondary
HTD40	DF	DV2	(3.88±0.45) ×10^5^	0.46	Neg	1.82	Pos	0.25	Secondary
HTD42	DF	DV1	(3.41±0.99) ×10^7^	0.77	Neg	2.49	Pos	0.31	Secondary
HTD45	DHF	DV1	(6.57±3.76) ×10^5^	0.51	Neg	3.61	Pos	0.14	Secondary
HTD49	DHF	DV2	(4.68±0.43) ×10^4^	1.25	Pos	1.49	Pos	0.84	Secondary
hD30	DHF	DV2	ND	4.54	Pos	6.59	Pos	0.69	Secondary

a ID, patient identification.

b Dengue disease was determined by examining the clinical symptoms of patients according to the WHO criteria.

c DENV serotype was determined by RT-PCR with universal and serotype-specific primers [31].

d Viremia titers were determined in a focus-forming immunoassay in semi-adherent K562 cells [30].

e IgM in patient serum was detected by Dengue Virus IgM Capture DxSelect. An index value of >1.00 was interpreted as positive (POS) and an index value of <1.00 was interpreted as negative (NEG).

f IgG in patient serum was detected by Dengue Virus IgG DxSelect. An index value of >1.00 was interpreted as positive (POS) and an index value of <1.00 was interpreted as negative (NEG).

g Cases with an IgM/IgG index ratio of ≤1.2 were diagnosed as secondary infections [32].

h Patient’s blood specimen was used for huMAb preparation as described elsewhere [24]. ND, not detectable.

We also assessed sera from four HTD patients for neutralization activity and ADE levels using antibodies from different patients infected with the same DENV serotype. Although we used heterologous sera from different patients, the antibodies neutralized heterologous virus infections at low serum dilutions (1Log_10_) and no ADE induction has been observed (Figure S1).

These results clearly demonstrate that antibodies present in the ten serum samples from DF and DHF patients infected with DENV-1, -2, or -3, were not able to induce serum-derived DENV infection enhancement in K562 cells. In addition, eight out of ten sera neutralized the major virus population with which the patient had been infected. Despite the fact that the samples contained polyclonal antibodies, these results show that the majority of antibodies induced by the infecting virus prevented the virus from infecting K562 cells.

### Laboratory-adapted strains of DENV show high levels of ADE and low neutralization activity in the presence of patient sera

**Figure 3:**
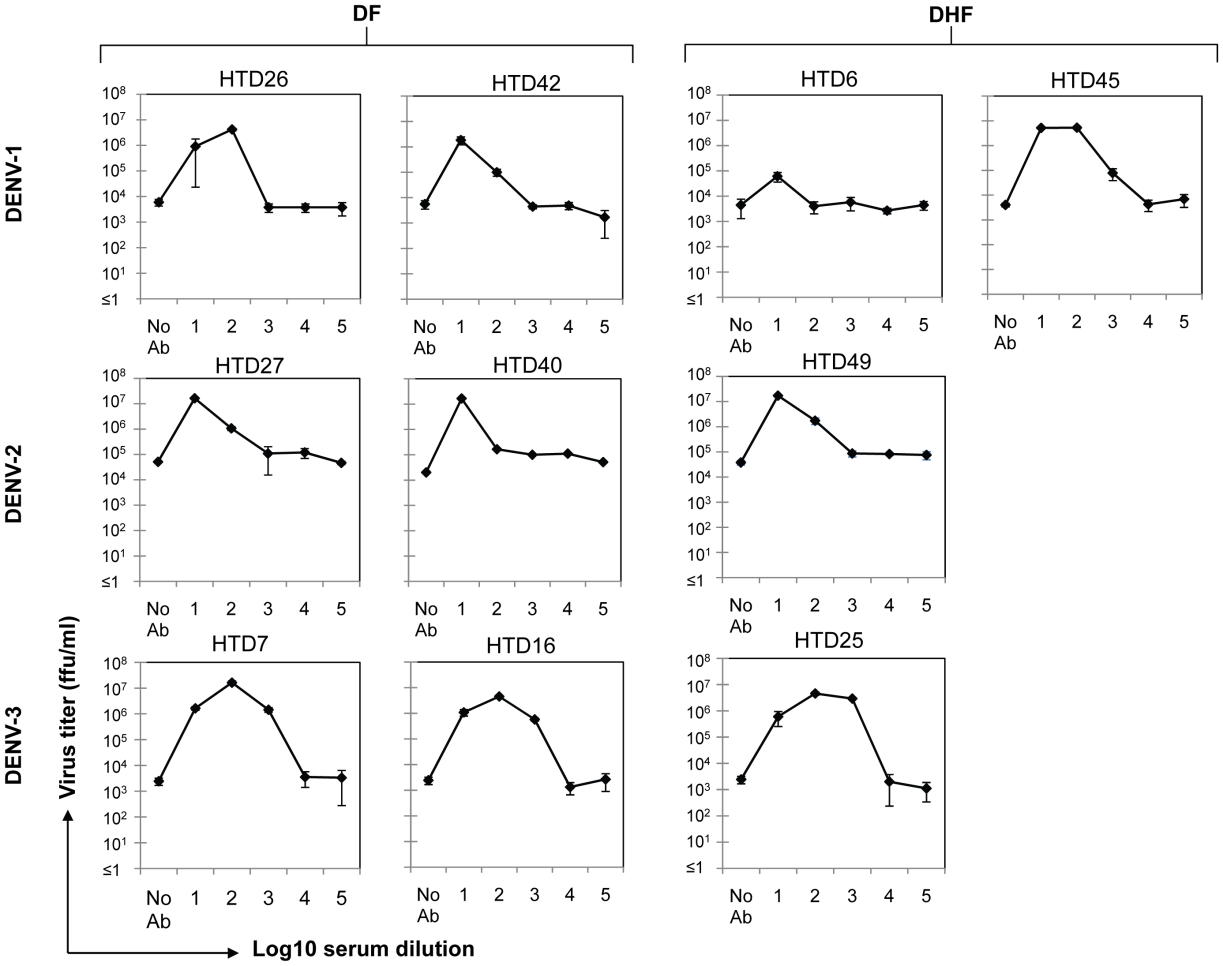
High levels of ADE when using patient plasma and laboratory-culture adapted DENV. Ultracentrifugation supernatants of patient sera were heat-inactivated at 56°C for 30 min, diluted 10-fold, and pre-mixed at an MOI of 0.02 with laboratory culture-adapted DENV-1, -2, and -3 for 30 minutes at 37°C. Virus-antibody complexes were added to K562 cells and incubated for 2 hours at 37°C. Maintenance medium supplemented with 2% FBS was then added before a further incubation for 3 days. Supernatants were harvested for virus titration in focus-forming immunoassays in Vero cells. Results are expressed as the mean ± SD of triplicate experiments. ‘No Ab’ means virus infection in the absence of plasma. The ‘No Ab’ value was used as a base line for calculating virus infection enhancement.

Previously, we reported that the majority of HuMAbs (prepared by fusing PBMC from dengue patients at the acute phase of a secondary DENV-2 infection with SPYMEG cells) showed ADE activity in assays employing THP-1 cells and the DENV-2 strain 16681 [23]. Here, we examined ADE activity using laboratory-adapted strains of DENV and patient sera as a source of anti-DENV antibodies (Figure 3). Serotypes of the laboratory-adapted strains used in the ADE assay were the same as those identified in the patients: DENV-1 strain 16007 for patients HTD26, HTD42, HTD6, and HTD45; DENV-2 strain 16681 for patients HTD27, HTD40, and HTD49; and DENV-3 strain 16562 for patients HTD7, HTD16, and HTD25. The K562 cells used for these ADE assays were the same as those used in Figure 2.

ADE activity induced by sera from patients in the presence of laboratory-adapted virus strains was greatly increased (Figure 3). Furthermore, no virus neutralization was detected when using patient-derived sera and laboratory-adapted strains of DENV. ADE was highest (around ×10,000) in the serum sample obtained from HTD7 (a DF patient infected with DENV-3). ADE levels for DF patients HTD26, HTD40, HTD16 and for DHF patients HTD45 and HTD25 were around ×1,000. ADE in serum samples obtained from HTD27, HTD42 and HTD49 was ×100, and that for HTD6 (a DHF patient) was around ×10. Taken together, these results revealed that there was no correlation between the results of neutralization/ADE assays and the type of disease manifestation (DF or DHF), or the DENV serotype that caused the disease.

### Replication kinetics of recombinant DENVs containing prM-E sequences obtained from virus samples of patients

Sequence variations in their prM-E encoding regions could be a reason for the observed differences in ADE and neutralization levels between serum-derived DENV and laboratory-adapted virus strains. Furthermore, serum-derived viruses might be present within immune complexes, which may render the viruses more sensitive to neutralization than laboratory-adapted strains. To test these hypotheses, we compared ADE levels induced by recombinant DENVs containing patient-derived prM-E gene sequences.

Using viral RNA extracted from the plasma of DHF patient D30 (D30-plasma), we constructed four infectious DENV cDNA clones harboring different amino acid substitutions within their prM-E region: R05/D30_MP, R05/D30_pr_P61T, R05/D30_E_M125I, and R05/D30_E_V365I. R05/D30_MP contained the prM-E gene sequence derived from a major DENV-2 population in D30-plasma (as was detected among 78.05% (32/41) of the clones). Other recombinant viruses with amino acid substitutions in the prM-E region were detected among 2.44% (1/41) of R05/D30_pr_P61T and R05/D30_E_M125I clones, among 4.88% (2/41) of the R05/D30_E_V365I clone, and among 12.19% (5/41) of the R05/D30_E_C302Y clone. Since we could not obtain a sufficient titer of R05/D30_E_C302Y, the remaining four recombinant viruses were assayed for levels of virus neutralization and ADE induction, and the levels were compared with those of parental R05-624 virus and DENV-2 strain 16681. A phylogenetic analysis of the D30 plasma-derived recombinant DENVs including the parental R05-624 clone, is shown in Figure S2. The five sequences of the prM-E region are closely related to each other and to those of Thai clinical isolates previously registered in the NCBI database.

**Figure 4:**
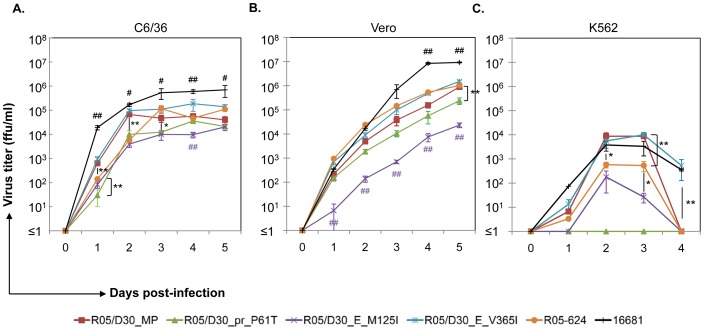
Replication kinetics of recombinant DENVs. DENV-2 16681 or individual recombinant DENVs were used to infect C6/36 (A) and Vero cells (B) at an MOI of 0.001, or K562 cells (C) at an MOI of 0.1. After 2 hours of incubation, the supernatants were removed and cells were washed twice with plain medium before the addition of maintenance medium supplemented with 2% FBS. For infected C6/36 and Vero cells, the supernatants were harvested daily. For infected K562 cells, both the culture medium and infected cells were harvested and centrifuged. Virus titers in the supernatants were determined in focus-forming assays in Vero cells. Results are expressed as mean ± SD of triplicate experiment (*p<0.05 and **p<0.01, unpaired two-tailed Student’s t-test, n = 3 per point). Statistically significant differences between data points are indicated by # (# *p<0.05*, ## *p<0.01*).

Prior to investigating the recombinant viruses in neutralization and ADE assays, we validated the infectivity of the molecular clones in several different host cells, including C6/36, Vero, and K562. As shown in Figure 4A, DENV-2 16681 produced a significantly higher level of progeny viruses than the recombinant viruses or the parental R05-624 strain at all time points (*p<0.05* at Days 2, 3 and 5, *p<0.01* at Days 1 and 4). The rate of replication and virus production among the recombinant viruses was statistically significantly different at each time point. For instance, the replication rates of R05/D30_MP and R05/D30_E_V365I were significantly higher than those of R05/D30_pr_P61T, R05/D30_ E_M125I and parental R05-624 strain during the first 2 days of infection (*p<0.01*). By contrast, the replication kinetics of DENV-2 strain 16681 in Vero cells were similar to those of all four recombinant viruses including the parental R05-624 strain during the first 3 days of virus infection, except on Days 4 and 5 post-infection (*p<0.01*). Although R05/D30_E_M125I showed similar replication kinetics, the overall level of virus production was significantly lower than that of the other strains (*p<0.01*; Figure 4B).

Virus production in the culture fluids of K562 cells differed between recombinant viruses and control viruses. The R05/D30_pr_P61T strain did not produce progeny viruses in K562 cells (Figure 4C). R05/D30_MP, R05/D30_E_V365I and DENV-2 16681 showed replication kinetics similar to those of R05/D30_E_M125I and the parental R05-624 strain; however, overall virus production of the latter two was significantly lower, especially at Days 2 (*p<0.05*) and 3 (*p<0.01*) post-infection. In addition, the recombinant viruses, as well as the parental R05-624 virus and DENV-2 16681 showed declining virus production levels in K562 cells at 4 days post-infection, a time point at which virus production was still increasing in C6/36 and Vero cells.

### Virus neutralization and ADE levels of recombinant DENVs are affected by HuMAb

**Figure 5:**
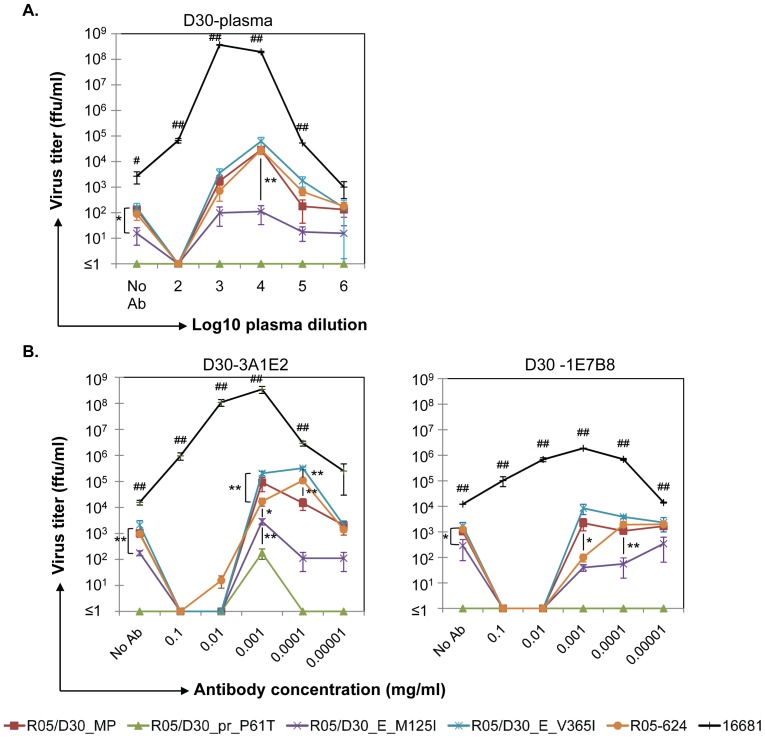
ADE levels of recombinant DENVs or DENV-2 strain 16681 exposed to D30-plasma or HuMAbs. A heat-inactivated D30-plasma sample (A) or purified human MAbs derived from patient D30 (B) were serially diluted 10-fold in RPMI-1640 medium and incubated with either DENV-2 16681 or individual recombinant DENVs for 30 min at an MOI of 0.1. Virus-antibody complexes were added to K562 cells and incubated for a further 2 h; maintenance medium was then added (without washing the cells) to yield a FBS final concentration of 2%. Cells and supernatants were collected on Day 3 post-infection. Virus titers in the supernatants were determined in focus-forming immunoassays in Vero cells. Results are expressed as the mean ± SD from two independent experiments performed in triplicate (*p<0.05 and **p<0.01, unpaired two-tailed Student t-test, n = 3 per point). Statistically significant differences between data points are indicated by # (# *p<0.05*, ## *p<0.01*).

Virus neutralization and ADE assays were performed in K562 cells using recombinant viruses and DENV-2 strain 16681. Similar to the results shown in Figure 3, replication of the DENV-2 strain 16681 was strongly enhanced, as shown by 10^5^-fold increases in the peak enhancement titer (PENT); however, the virus was resistant to neutralization (Figure 5A, Supplemental Figure S3A). Also, the virus titer of DENV-2 16681 was significantly higher than that of other viruses when serum was added at 2Log_10_ to 5Log_10_ dilutions (*p<0.01*; Figure 5A). Conversely, the parental R05-624 virus and all D30-derived recombinant viruses (except R05/D30_pr_P61T) were positive for both virus neutralization and ADE activity. Interestingly, the titers of the recombinant viruses in the absence of antibodies were only 10-30 fold higher than that of R05/D30_E_M125I (*p<0.05*), whereas the virus titers at the PENT were 260-560 fold higher than that of R05/D30_E_M125I (*p<0.01*).

Antibody preparations from individual patient plasma would be expected to contain polyclonal antibodies that recognize different virion surface epitopes; thus they would be expected to react differently with the viral population(s) used in the assays. To analyze whether the polyclonal nature of antibodies in patient plasma was responsible for our observations, we used two HuMAbs previously prepared from PBMC isolated from the same DHF patient, D30 [23].

As shown in Figure 5 and S3, the viruses that showed the highest and lowest ADE (R05/D30_E_V365I and R05/D30_E_M125I, respectively) yielded similar results when either D30-derived plasma (Figure 5A) or a D30-derived HuMAb (D30-3A1E2) were used in the assay (Figure 5B). Interestingly, HuMAb D30-3A1E2, but not D30-plasma, increased the production of R05/D30_pr_P61T ∼ 7-fold. On the other hand, another D30-derived HuMAb, D30-1E7B8, showed strong neutralization activity against all of the cloned viruses and against the parental R05-624 strain. However, it showed weak ADE against the D30-derived recombinant viruses and the parental R05-624 strain, and weaker ADE activity than HuMAb D30-3A1E2 in presence of DENV-2 16681.

Although R05/D30_MP, R05/D30_E_V365I, and the control DENV-2 strain 16681 showed similar virus production levels in K562 cells (Figure 4C), PENT for DENV-2 16681 was significant higher (1.38×10^5^ fold) than that of the other two strains (2.17×10^2^ fold for R05/D30_MP and 4×10^2^ fold for R05/D30_E_V365I; *p<0.01*; Supplemental Figure S3A). The level of ADE (3.25×10^2^ fold) in the presence of the parental R05-624 virus was not different from that in the presence of the other two virus variants, R05/D30_MP and R05/D30_E_V365I (Figure 5), although overall virus production was significantly lower (*p<0.01*; Figure 4C). The PENT for all infectious clones, including parental R05-624, occurred at a plasma dilution of 4Log_10_, while the plasma dilution peak for DENV-2 16681 was 3Log_10_ (Figure 5A). When the DENV-2 strain 16681 was assayed, virus production increased by more than 10^4^-fold over that of the control (no anti-DENV antibodies) in the presence of 0.001 mg/ml HuMAb D30-3A1E2. (Figure 5B and Figure S3B, left panel). Also, the PENT for R05/D30_E_V365I and the parental R05-624 strain occurred in the presence of 0.0001 mg/ml HuMAb D30-3A1E2 (Figure 5B and Figure S3B, right panel). Nevertheless, the PENT for D30 E_M125I was significantly lower than that of other viruses when either plasma or HuMAb were used (*p<0.01*; Figure S3).

Data from our previous paper showed that HuMAb D30-1E7B8 reacts with the full-length E protein, but not with truncated forms of E, indicating that this HuMAb recognizes a conformational epitope [23]. This HuMAb neutralized or blocked all virus replication at a concentration of 0.01–0.1 mg/ml (Figure 5B, right panel); however, it enhanced DENV-2 strain 16681 infection by approximately 100-fold at a lower concentration (0.001 mg/ml).

While other studies [21], [23], [30] used virus titers assessed in focus-forming immunoassays in Vero cells to estimate the MOI, our data demonstrated that virus titers assessed in K562 cells were more favorable to estimate optimal MOI for replication kinetics in K562 cells and ADE assays than Vero cells. When using virus titers assessed in K562 cells to estimate MOI for virus infection, we found that replication rates and virus production levels in K562 cells of recombinant DENVs, parental R05-624 strain and DENV-2 16681 were significantly higher than those when assessed in Vero cells (*p<0.05* at Days 2 and *p<0.01* at Days 3 post-infection; Supplemental Figure S4A). Furthermore, optimal MOI estimated from virus titers assessed in K562 cells resulted in statistically significantly higher virus titers at PENT of all viruses (*p<0.05*), except R05/D30_pr_P61T (Figure S4B).

### Comparison of K562 and THP-1 cells for ADE activity measurement

Previous studies assayed ADE in K562 [10], [26], [34], THP-1 [23], [35]–[37] or BHK [38], [39] cells. Therefore, we used both THP-1 and K562 cells to assay ADE (Supplemental Figure S5). D30-plasma was serially diluted 10-fold and mixed with the D30-derived recombinant viruses, the parental R05-624 virus, and the DENV-2 strain 16681 at an MOI of 0.1 for each virus. After incubation for 30 min, THP-1 cells were infected with the mixture. On day 3 post-infection, total RNA extracted from the cell fraction was subjected to quantitative RT-PCR (Supplemental Figure S5A). The fluid fraction was titrated in a focus-forming immunoassay in Vero cells (Supplemental Figure S5B). High levels of ADE activity were observed for DENV-2 16681 (*p<0.05* for fold enhancement and *p<0.01* for virus titer), but not for the other recombinant DENVs (Supplemental Figure S5A and S5B). The same virus and antibody samples were then subjected to ADE assays in K562 cells under the conditions shown in Figure S4B (Supplemental Figure S5C). Again, DENV-2 16681 showed high level of ADE, whereas the D30-derived recombinant viruses showed only low ADE levels.

## Discussion

This study examines virus neutralization and ADE activity *in vitro* using the K562 cell line along with polyclonal antibodies and DENV derived from the serum of dengue patient during the acute phase of a secondary DENV infection. The data demonstrate that serum from patients showed high levels of virus neutralization and low levels of ADE activity in the presence of viruses isolated from the same patients, whereas there was no neutralization and high levels of ADE activity in the presence of serotype-matched laboratory strains. Thus, the source of the virus had a profound effect on virus neutralization and ADE. We showed that DENV derived from patient sera were susceptible to neutralization but exhibited no ADE activity when pre-incubated with autologous serum. In most of these cases, the serum had opposing effects when assayed using laboratory strains of DENV matched to the serotype isolated from the patient. This difference could be due to unidentified viral factor(s), as well as to the properties of anti-DENV antibodies present in the patient serum.

The K562 cell line is often used to assay the ADE activity of DENV because it expresses activating FcγRIIa receptors but not inhibitory FcγRIIb receptors [34], [40]–[42]. Complement was excluded from our system by heating plasma at 56°C for 30 min before the assay. Hence the observed virus neutralization could only be due to the blocking of DENV replication by the specific antibodies. As DENV-containing immune complexes have been detected in patient plasma [43]–[45], the virus fractions used in our experiments may have contained virus-antibody complexes that augment virus replication in K562 in the absence of serum antibodies. However, the results reported herein show that addition of the antibody fraction of autologous serum at a 1Log_10_ dilution diminished or blocked the infection in K562 cells by virus-antibody complexes by 10-fold to 1000-fold.

Most studies on ADE indicate that human sera obtained from patients with a secondary DENV infection possess the ability to enhance infection of cell lines by laboratory-adapted DENV *in vitro* [23], [46], [47]. Some studies used clinical isolates prepared from dengue patient plasma, which had been passaged in cell lines [38], [48]. Therefore, we used laboratory-grown DENV with a serotype identical to that of the serotype detected in patient plasma. The results were in agreement with other findings: all ten sera from dengue patients with secondary infections enhanced antibody-dependent DENV infection of K562 cells. As seen in other studies [23], [38], [47], we also found that there was no correlation between *in vitro* ADE activity and disease severity. In the present study, only one out of ten serum samples (HTD45) was incapable of virus neutralization. Further investigations using more serum samples are necessary to confirm and extend these findings.

When using antibodies derived from patient serum, we found that the neutralization and ADE activities when assayed with autologous viruses were contrary to those when using serotype-matched laboratory strains. This may be due to considerable prM-E amino acid sequence diversity between serum-derived viruses and laboratory strains. Due to the nature of viral quasi-species, viruses derived from an individual patient’s serum or plasma comprise a mixture of variants. Each of these variants would be expected to respond differently to an anti-DENV antibody obtained from autologous serum or plasma. Laboratory-adapted viruses are usually selected for their ability to grow *in vitro* and may have adapted to serial passage in tissue culture. To test this hypothesis, we utilized recombinant DENV containing the prM-E region derived from virus populations in plasma of DHF patient D30 to investigate whether amino acid substitutions in this region can affect ADE. ADE was higher in the presence of the DENV-2 16681 control virus than in the presence of any of the viral clones; this was true irrespectively of whether autologous plasma or HuMAb were used in the assay. The nucleotide sequence homology between the prM-E region of the DENV-2 16681 and that of the major DENV-2 population in D30-plasma, and between DENV-2 16681 and the parental R05-624 virus were 97.2 and 96.9%, respectively. The possibility that one or more nucleotide changes in the viral genome, particularly in the prM-E encoding region of the laboratory-adapted virus produced an “enhancing epitope” requires further investigation.

Another explanation for the differences in ADE activity between patient-derived and laboratory-adapted strains of DENV could be that serum-derived antibodies bind to virions as a complex; thus they are found in the precipitate fraction of plasma. Such bound antibodies may play a role in virus neutralization and would be absent from assays using laboratory strains. Therefore, we examined the neutralization and ADE activity of recombinant DENVs containing the prM-E region of DENV2 from patient D30 to ensure the absence of any immune complexes. Interestingly, the parental R05-624 virus and all the D30-derived cloned viruses were positive for both virus neutralization and ADE activity. We observed high levels of virus neutralization at high antibody concentrations when using plasmid-derived virus variants and the parental R05-625. When we used D30-plasma and HuMAb-D30-3A1E2, virus infection increased by 10-fold to 100-fold at low antibody concentrations. These results concur with other findings, i.e., that ADE can occur in the presence of neutralizing antibodies, but only at very low or sub-neutralizing antibody concentrations [7], [23], [49]. These plasmid-derived viruses, although identical in sequence to viruses detected in patient plasma, had been passaged once in C6/36 cells, which could have introduced mutations in their genomes outside prM-E affecting ADE.

Although the levels of ADE induced by D30-plasma or HuMAb D30-3A1E2 were comparable, HuMAb D30-1E7B8 exhibited strong neutralizing activity but did not induce ADE activity when assayed in the presence of recombinant DENVs. However, this antibody increased DENV-2 16681 replication by 100-fold. These results suggest that ADE and neutralization of DENV are the overall outcome of a combination of diverse DENV variants and their specific antibodies. As shown in Figure 5, HuMAb D30-3A1E2 was likely the main antibody in D30-plasma since it elicited a similar pattern of ADE induction to the D30-plasma. This ADE effect could overcome the strong neutralizing ability of HuMAb D30-1E7B8.

In conclusion, this study demonstrates that DENVs derived from patient serum elicit patterns of ADE that are different from those of laboratory-grown DENVs. Therefore, it is important to be careful when interpreting the enhancing or neutralizing effects of antibodies when using different sources of DENV. Furthermore, we also demonstrate that a single amino acid substitution in E, V365I, can affect ADE. However, the mechanism by which this amino acid position influences ADE requires further investigation.

## Supporting Information

Figure S1 **Comparison of ADE among heterologous DENV isolates of the same serotype.** Serum samples (DENV-1, DENV-2, and DENV-3) from HTD dengue patients (three with DF and one with DHF clinical manifestations) were ultracentrifuged to precipitate the DENV virions. The supernatant fractions were heat-inactivated at 56°C for 30 minutes, and then serially diluted 10-fold. The dilutions were mixed with serum-derived viruses from the precipitate fraction of autologous plasma for 30 minutes at 37°C at an MOI of 0.02. The virus-antibody complexes were added to K562 cells and incubated for 2 hours at 37°C before the addition of maintenance medium supplemented with 2% FBS. The cells were then incubated for a further 3 days. Supernatants were harvested for virus titration in a focus-forming immunoassay in Vero cells. The results are expressed as FFU/ml. The mean ± SD of triplicate experiments is shown. ‘No Ab’ means virus infection in the absence of plasma. ‘No Ab” values were used as a baseline for calculating virus infection enhancement. **Ab** in bold type indicates antibody from a DHF patient. (*p<0.05 and **p<0.01, unpaired two-tailed Student’s t-test, n = 3 per point). # indicates a statistically significant difference between one specific point and the others (# *p<0.05*, ## *p<0.01*).
(TIF)

Figure S2 **Phylogenetic tree of the prM-E encoding region of the infectious molecular clones.** The phylogenetic tree was based on the nucleotide sequences of the prM-E region of the molecular clones derived from patient D30, the parental clone R05-624 and sequences from the NCBI database that were registered as the DENV-2 “Asian I” genotype from Thailand. The phylogenetic tree was generated using the Maximum Likelihood algorithm based on the Tamura-Nei model with bootstrapping (500 iterations). Evolutionary analyses were conducted in MEGA5.
(TIF)

Figure S3 **Peak enhancing titers (PENT) of recombinant DENVs.** Fold-enhancement was calculated from the virus titer data in Figure 5 by dividing the average number of foci at the highest virus titer in the presence of antibody by the average number of foci in the absence of antibody. Results are expressed as mean ± SD from two independent experiments performed in triplicate. (*p<0.05 and **p<0.01, unpaired two-tailed Student’s t-test, n = 3 per point). Statistically significant differences between specific points and the others are indicated by # (# *p<0.05*, ## *p<0.01*). n.s., not significant.
(TIF)

Figure S4 **Replication kinetics and ADE of recombinant DENVs in K562 cells.** (A) Virus titers of the recombinant viruses, parental strain R05-624, and DENV-2 16681 were assessed in a focus-forming immunoassay in Vero cells and K562 cells to estimate optimal MOI. K562 cells were infected with DENV at an MOI of 0.1 after virus titration in Vero (dashed lines) or K562 cells (solid lines). After incubation at 37°C for 2 hours, viruses were removed and the infected cells were washed before the addition of maintenance medium supplement with 2% FBS. Supernatant were harvested consecutively within 4 days for virus titration in focus-forming immunoassay in Vero cells. Results are expressed as the mean ± SD of triplicate experiments. (B) For the ADE assay, 10-fold dilutions of heat-inactivated D30-plasma were pre-incubated with the recombinant virus variants, the parental R05-624 strain, or DENV-2 16681 at an MOI of 0.1 (which was estimated from virus titers assessed in Vero (solid bars) and K562 cells (open bars)). Then, the virus-antibody complexes were added to K562 cells and incubated for a further 2 h. Maintenance medium was added to yield a final FBS concentration of 2%. Cells and supernatants were collected on Day 3 post-infection. Virus titers in the supernatants were determined in a focus-forming immunoassay in Vero cells. Fold-enhancement was calculated by dividing the average number of foci at the highest virus titer in the presence of antibody by the average number of foci in the absence of antibody. Results are expressed as the mean ± SD from two independent experiments performed in triplicate. (*p<0.05 and **p<0.01, unpaired two-tailed Student’s t-test, n = 3 per point).
(TIF)

Figure S5 **ADE assays for recombinant DENVs in THP-1 and K562 cells (**A) Heat-inactivated D30-plasma was serially diluted 10-fold in medium, and the dilutions were incubated at an MOI of 0.1 with recombinant DENVs derived from patient D30 plasma. Virus-antibody complexes were incubated with THP-1 cells for another 2 hours before adding maintenance medium. On day 3 post-infection, cells were harvested and DENV replication analyzed via one-step quantitative RT-PCR. The virus-antibody complexes were added to K562 cells and treated as described for THP-1 cells. On day 3 post-infection, the culture fluids from the samples in Figure A (B) and the infected K562 cells (C) were collected and their titers were determined in a focus-forming immunoassay in Vero cells. Results are expressed as the mean ± SD of triplicate experiments.
(TIF)
