# Supplementary figures and images for: Low Levels of Antibody-Dependent Enhancement in Vitro Using Viruses and Plasma from Dengue Patients
Source: PLoS One. 2014 Mar 18;9(3):e92173. doi: 10.1371/journal.pone.0092173 (PMC3958444; doi:10.1371/journal.pone.0092173)

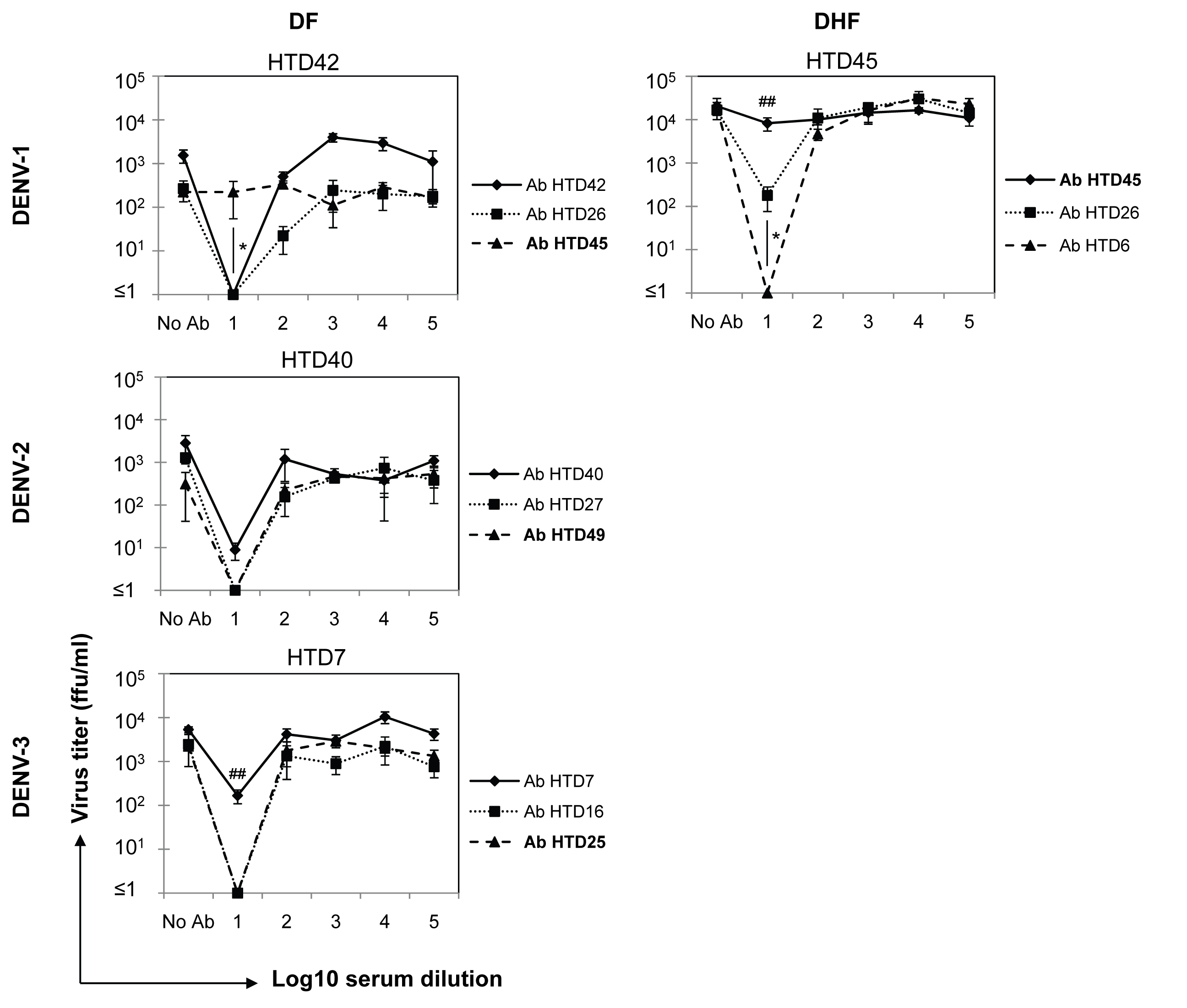

Supplement: Figure S1 — Comparison of ADE among heterologous DENV isolates of the same serotype. Serum samples (DENV-1, DENV-2, and DENV-3) from HTD dengue patients (three with DF and one with DHF clinical manifestations) were ultracentrifuged to precipitate the DENV virions. The supernatant fractions were heat-inactivated at 56°C for 30 minutes, and then serially diluted 10-fold. The dilutions were mixed with serum-derived viruses from the precipitate fraction of autologous plasma for 30 minutes at 37°C at an MOI of 0.02. The virus-antibody complexes were added to K562 cells and incubated for 2 hours at 37°C before the addition of maintenance medium supplemented with 2% FBS. The cells were then incubated for a further 3 days. Supernatants were harvested for virus titration in a focus-forming immunoassay in Vero cells. The results are expressed as FFU/ml. The mean ± SD of triplicate experiments is shown. ‘No Ab’ means virus infection in the absence of plasma. ‘No Ab” values were used as a baseline for calculating virus infection enhancement. Ab in bold type indicates antibody from a DHF patient. (*p<0.05 and **p<0.01, unpaired two-tailed Student’s t-test, n = 3 per point). # indicates a statistically significant difference between one specific point and the others (# p<0.05, ## p<0.01). (TIF) [file pone.0092173.s001.tif]

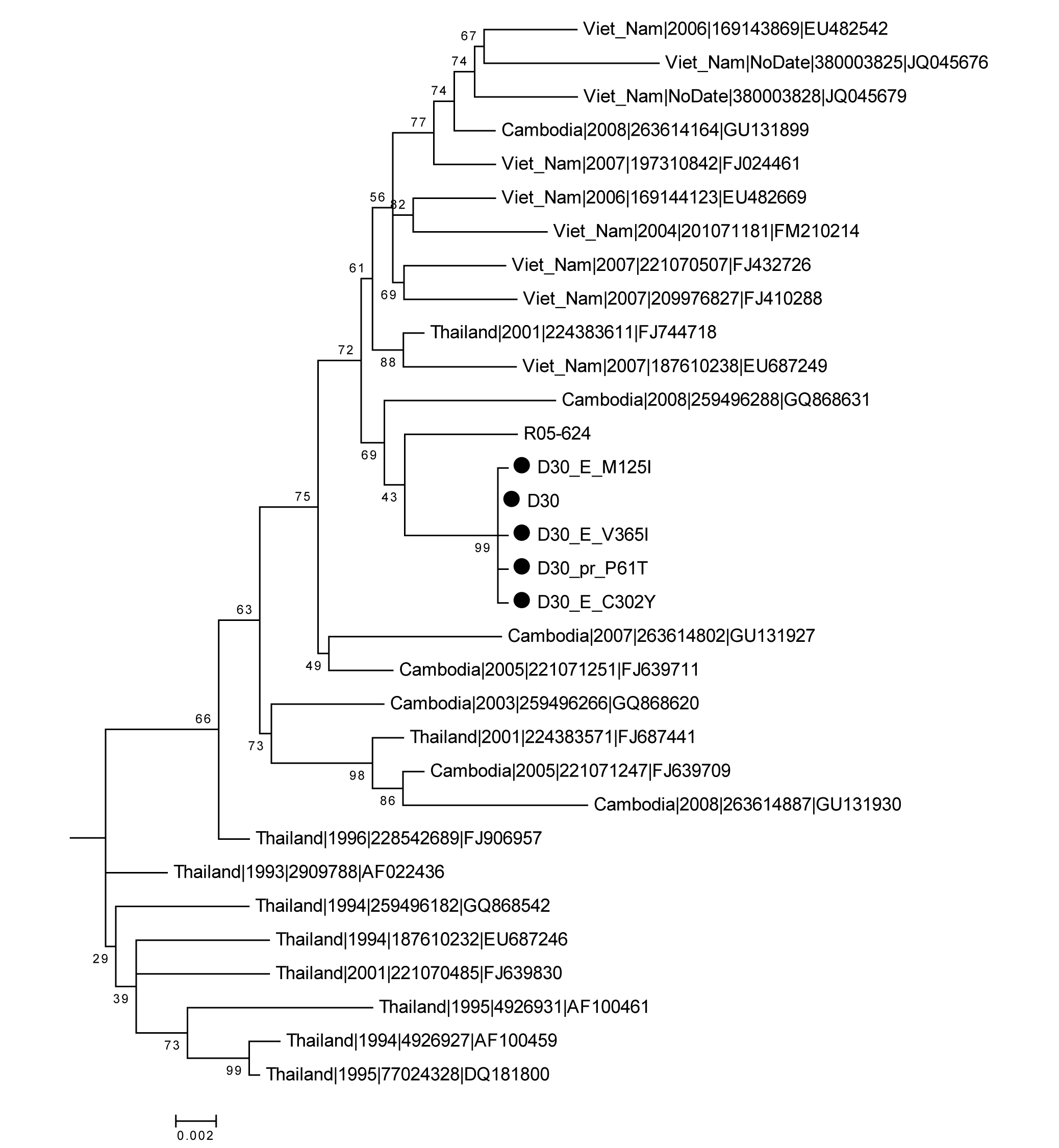

Supplement: Figure S2 — Phylogenetic tree of the prM-E encoding region of the infectious molecular clones. The phylogenetic tree was based on the nucleotide sequences of the prM-E region of the molecular clones derived from patient D30, the parental clone R05-624 and sequences from the NCBI database that were registered as the DENV-2 “Asian I” genotype from Thailand. The phylogenetic tree was generated using the Maximum Likelihood algorithm based on the Tamura-Nei model with bootstrapping (500 iterations). Evolutionary analyses were conducted in MEGA5. (TIF) [file pone.0092173.s002.tif]

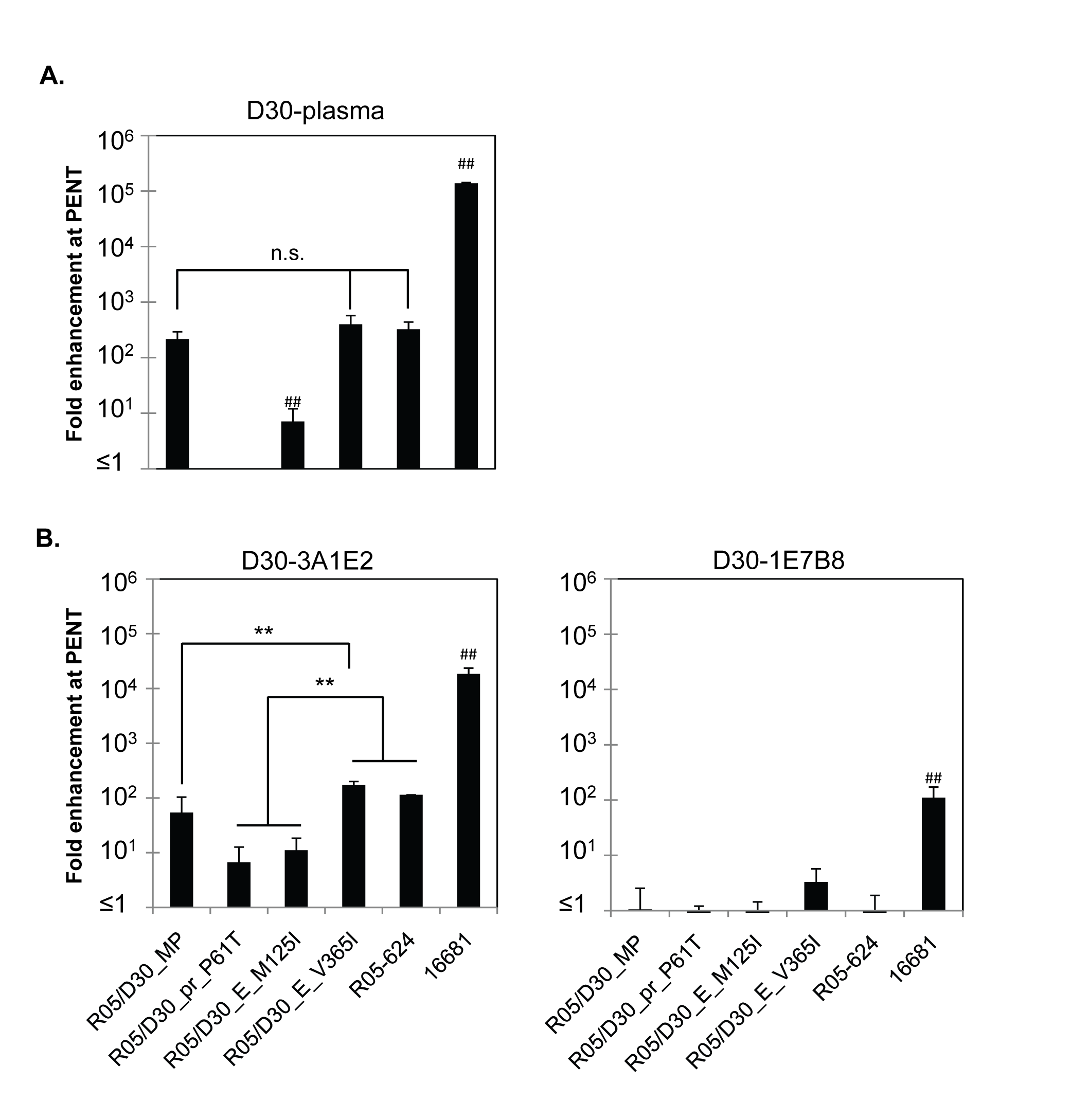

Supplement: Figure S3 — Peak enhancing titers (PENT) of recombinant DENVs. Fold-enhancement was calculated from the virus titer data in Figure 5 by dividing the average number of foci at the highest virus titer in the presence of antibody by the average number of foci in the absence of antibody. Results are expressed as mean ± SD from two independent experiments performed in triplicate. (*p<0.05 and **p<0.01, unpaired two-tailed Student’s t-test, n = 3 per point). Statistically significant differences between specific points and the others are indicated by # (# p<0.05, ## p<0.01). n.s., not significant. (TIF) [file pone.0092173.s003.tif]

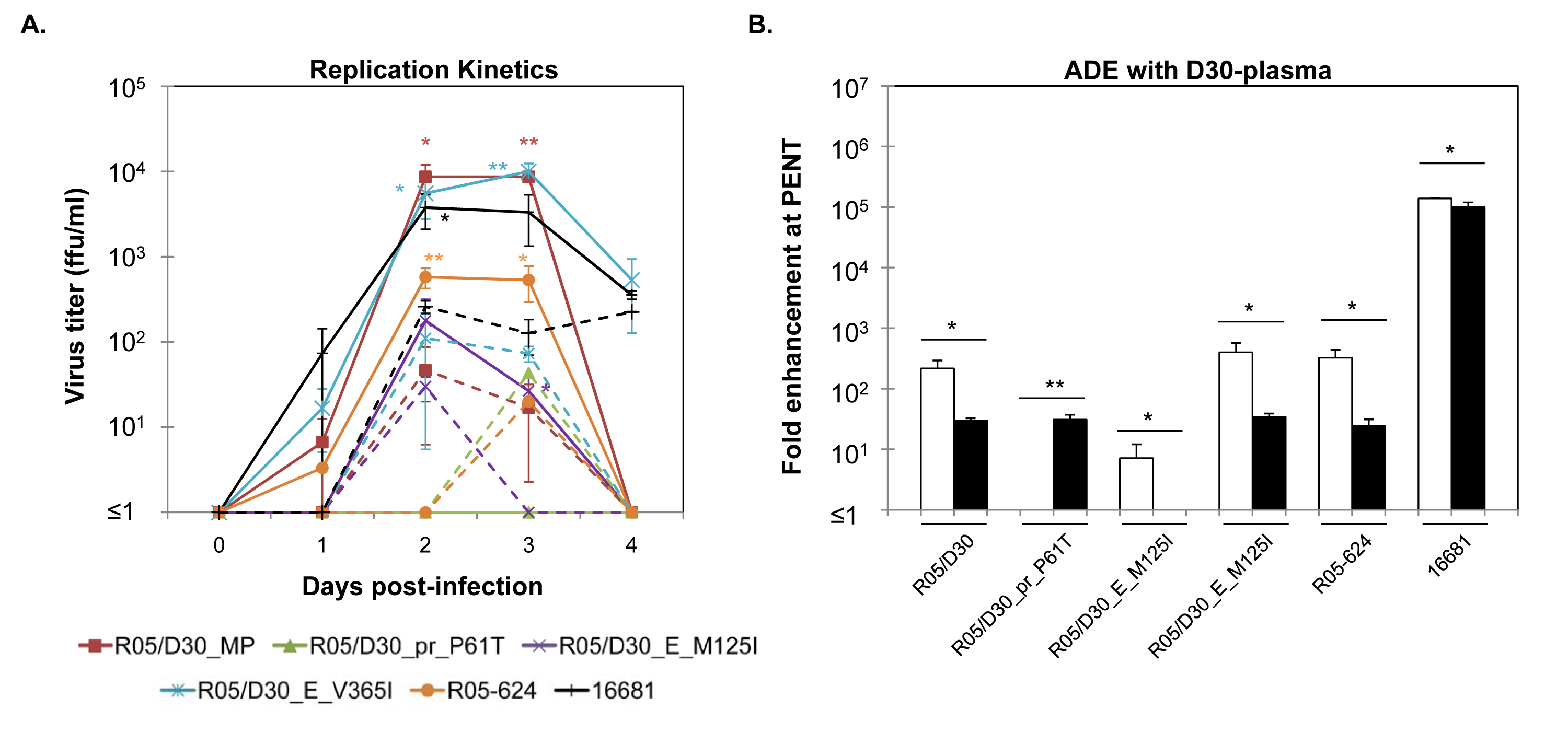

Supplement: Figure S4 — Replication kinetics and ADE of recombinant DENVs in K562 cells. (A) Virus titers of the recombinant viruses, parental strain R05-624, and DENV-2 16681 were assessed in a focus-forming immunoassay in Vero cells and K562 cells to estimate optimal MOI. K562 cells were infected with DENV at an MOI of 0.1 after virus titration in Vero (dashed lines) or K562 cells (solid lines). After incubation at 37°C for 2 hours, viruses were removed and the infected cells were washed before the addition of maintenance medium supplement with 2% FBS. Supernatant were harvested consecutively within 4 days for virus titration in focus-forming immunoassay in Vero cells. Results are expressed as the mean ± SD of triplicate experiments. (B) For the ADE assay, 10-fold dilutions of heat-inactivated D30-plasma were pre-incubated with the recombinant virus variants, the parental R05-624 strain, or DENV-2 16681 at an MOI of 0.1 (which was estimated from virus titers assessed in Vero (solid bars) and K562 cells (open bars)). Then, the virus-antibody complexes were added to K562 cells and incubated for a further 2 h. Maintenance medium was added to yield a final FBS concentration of 2%. Cells and supernatants were collected on Day 3 post-infection. Virus titers in the supernatants were determined in a focus-forming immunoassay in Vero cells. Fold-enhancement was calculated by dividing the average number of foci at the highest virus titer in the presence of antibody by the average number of foci in the absence of antibody. Results are expressed as the mean ± SD from two independent experiments performed in triplicate. (*p<0.05 and **p<0.01, unpaired two-tailed Student’s t-test, n = 3 per point). (TIF) [file pone.0092173.s004.tif]

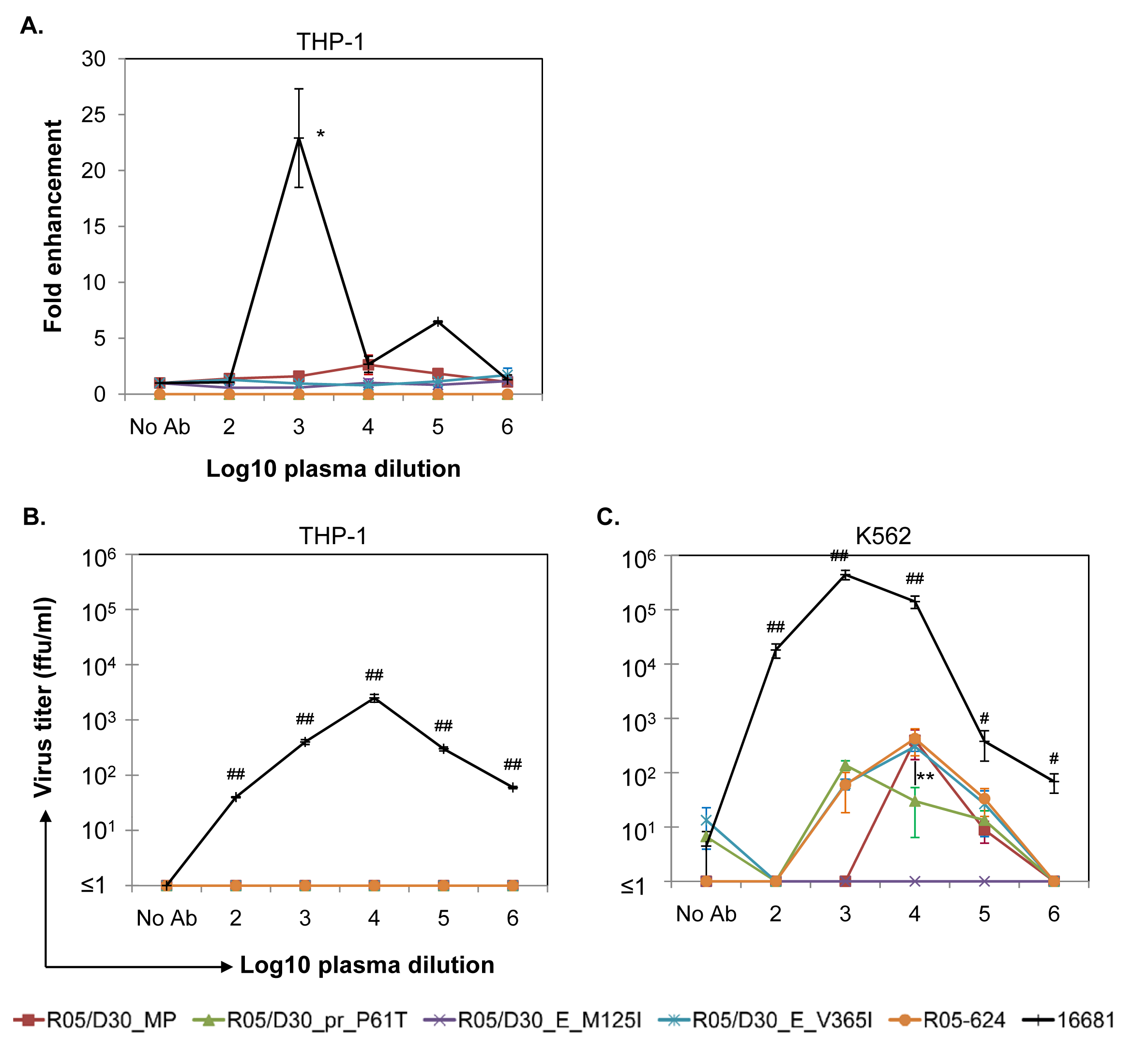

Supplement: Figure S5 — ADE assays for recombinant DENVs in THP-1 and K562 cells (A) Heat-inactivated D30-plasma was serially diluted 10-fold in medium, and the dilutions were incubated at an MOI of 0.1 with recombinant DENVs derived from patient D30 plasma. Virus-antibody complexes were incubated with THP-1 cells for another 2 hours before adding maintenance medium. On day 3 post-infection, cells were harvested and DENV replication analyzed via one-step quantitative RT-PCR. The virus-antibody complexes were added to K562 cells and treated as described for THP-1 cells. On day 3 post-infection, the culture fluids from the samples in Figure A (B) and the infected K562 cells (C) were collected and their titers were determined in a focus-forming immunoassay in Vero cells. Results are expressed as the mean ± SD of triplicate experiments. (TIF) [file pone.0092173.s005.tif]
